# Beyond Imitation: How Food Colloids Are Shaping the Next Generation of Biomimetic Foods

**DOI:** 10.3390/gels11030155

**Published:** 2025-02-20

**Authors:** Yong Guo, Jiacheng Wang, Lianxin Du, Chao Ma, Yan Xu, Xin Yang

**Affiliations:** 1 College of Sports and Human Sciences, Harbin Sport University, Harbin 150008, China; guoyong@hrbipe.edu.cn; 2 Medical College, Yangzhou University, Yangzhou 225009, China; jcw@yzu.edu.cn; 3 Graduate School, Harbin Sport University, Harbin 150008, China; dulianxin@hrbipe.edu.cn; 4 School of Medicine and Health, Harbin Institute of Technology, Harbin 150001, China; mmmmachao1996@163.com

**Keywords:** food colloids, biomimetic food, biomimetic, plant foods

## Abstract

In the new global landscape of population, environmental, and energy sustainability, the manufacture of future food products that meet human nutritional and health needs is a major challenge. Biomimetic food, as a new type of food, has made significant progress in the use of plant proteins and other ingredients to mimic animal food, and it has also achieved important results in sensory and nutritional properties. In the study of biomimetic foods, food colloids play an irreplaceable role as the key framework for building food structures. In this paper, we first review the recent research progress on food colloids in the fields of biomimetic plant-based food, biomimetic animal-based food and 3D printed biomimetic food. Then, the mechanism of action, application effects, and quality improvement strategies of food colloids are deeply analyzed. Finally, the future research directions and application prospects are envisioned. This paper aims to give theoretical support and practical guidance for the development of biomimetic food through the above elaboration, to deal with the current problems in food development by means of the unique properties of food colloids, and to open up new ideas for the application of food colloids in future food innovation, and then to promote the further development of the field of biomimetic food.

## 1. Introduction

The food industry is confronting unparalleled challenges driven by globalization and industrialization, including the sustainable use of resources, ensuring food safety, and meeting the growing consumer demand for healthy food options. In response to these challenges, biomimetic food has emerged as a promising solution, garnering significant attention from both industry and academia due to its superior nutritional, sensory, and processing properties. Biomimetic food represents a novel category of food products that mimic the sensory, nutritional, and processing characteristics of natural food while incorporating optimized composition, structure, and preparation processes. By improving nutritional value, enhancing sensory experiences, and optimizing processing performance, biomimetic food offers distinct advantages over traditional food products, positioning itself as a crucial development direction for the future of the food industry [1,2].

Central to the development of biomimetic food is the application of food colloids, which play a pivotal role in determining the texture, stability, and sensory properties of various food systems [3,4]. The combination of starch and xanthan gum thickens and enhances the stable sensory and texture characteristics of strawberry jam [5]. The hydrophilic colloid mixture of soybean flour and oat bran can improve the nutritional value of foods by reducing saturated fat content and increasing soluble fiber content [6]. The unique ability of food colloids to enhance sensory characteristics, improve stability, and boost nutritional value has made them an indispensable component in the design of biomimetic food and a key driver of innovative food development. The potential of food colloids to address major challenges faced by the food industry, such as creating healthier, more sustainable, and appealing food choices for consumers, has fueled a widespread interest in their application within biomimetic food research [7,8,9]. However, despite the extensive application prospects of food colloids, several controversies and challenges persist, particularly in terms of biocompatibility, environmental impact, and production costs. Moreover, as consumers increasingly demand transparency in food ingredients, concerns regarding the source and safety of food colloids have also come to the forefront. To address these issues and fully harness the potential of food colloids in biomimetic food design, in-depth research is crucial. Such research will not only drive innovation within the food industry but also hold significant implications for meeting evolving consumer demands and promoting environmental sustainability.

The intersection and integration of food colloid science and biomimetic food research have become increasingly intertwined in recent years, leading to the emergence of numerous innovative biomimetic food products that have significantly contributed to the advancement of the modern food industry. Notable achievements include plant protein-based biomimetic foods, composite gel-based biomimetic meat products (meat analogs made from plant protein), biomimetic dairy products utilizing emulsification and microencapsulation technologies, and personalized biomimetic foods created through 3D printing technology [10,11,12,13]. These research outcomes highlight the immense potential and extensive prospects of food colloid science in addressing the challenges associated with biomimetic food research and development, as well as in creating novel biomimetic food products.

This article builds upon the foundation of previous research to provide a comprehensive review of the latest progress in food colloid science within the realm of biomimetic food research. The primary focus will be on examining the applications of food colloid structure and function across three categories: plant-based foods, animal-based foods, and 3D-printed biomimetic foods. Additionally, this article will explore future research directions and challenges within this field, aiming to offer fresh perspectives and valuable insights for the ongoing research and development of biomimetic foods.

## 2. The Application of Food Colloids in Biomimetic Plant-Based Foods

Plant-based foods have gained significant popularity among consumers due to their nutritional benefits and environmental friendliness. However, when compared to traditional animal-based foods, plant-based options still exhibit gaps in sensory qualities such as taste and texture. By rationally selecting and formulating different types of food colloids, the sensory quality, texture, stability, and nutritional value of plant-based foods can be significantly improved. The introduction of food colloids provides new ideas and methods for enhancing the sensory quality of plant protein-based foods. This section will focus on exploring the application of food colloids in biomimetic plant-based foods, mainly covering three aspects: plant protein-based food colloid systems, polysaccharide-based food colloid systems, and composite plant-based food colloid systems.

### 2.1. Plant Protein-Based Food Colloid Systems

The application of food colloids in biomimetic plant-based foods is primarily reflected in plant protein-based food colloid systems. Plant proteins have become essential raw materials in biomimetic plant-based food research due to their rich nutritional value and multifunctionality [14]. However, compared to traditional animal-based foods, plant-based foods exhibit differences in sensory quality and functional properties, despite plant proteins being high-quality dietary components [15,16]. With the increasing interest in sustainable and healthy food alternatives, plant proteins have garnered attention as viable substitutes for animal proteins. Utilizing food colloid principles to improve the functional properties of plant proteins is crucial for expanding the application of plant protein foods.

Studies have shown that constructing plant protein-based food colloid systems can enhance the mouthfeel, texture, and stability of foods. Saxena et al. demonstrated that specific processing techniques such as soaking, germination, and ultrasonic treatment can improve the nutritional value and sensory quality of millet milk, providing an effective approach for developing plant-based dairy products [17]. Yang et al. compared pea protein-based yogurt and mung bean protein-based yogurt produced by fermentation, finding that mung bean protein-based yogurt exhibited better performance in hardness, chewiness, water-holding capacity, lightness, and storage modulus. Mung bean protein can serve as an alternative option for preparing plant-based yogurt, offering improved quality and mouthfeel [18]. Wang et al. showed that stable emulsions and oleogels can be prepared by the acid–heat treatment of peanut isolated protein, and this method can be utilized to develop new plant protein-based fat substitutes [19]. The use of plant-based alternatives in foods like ice cream offers the potential for improved physicochemical properties and additional health benefits [20].

The development of plant-based foods must consider both sensory and non-sensory attributes to meet consumer demands for personalized healthy foods. Despite the improvements in physicochemical properties and health benefits, plant-based alternatives still face challenges in terms of nutritional quality, anti-nutritional factors, and sensory perception. These issues affect consumer acceptance of these foods and highlight the areas that future research and product development need to address. In conclusion, the utilization of plant proteins plays a crucial role in driving the food industry towards a healthier and more sustainable direction while providing consumers with more diverse and personalized food choices.

### 2.2. Polysaccharide-Based Food Gel Systems

**Figure 1:**
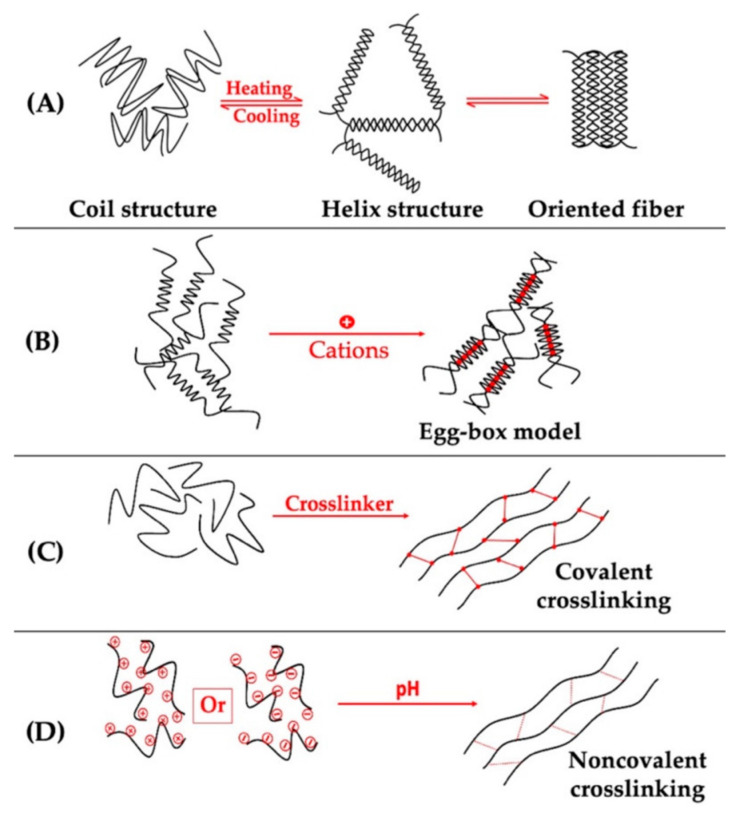
Illustration of the main mechanisms of formation of polysaccharides. (**A**) Temperature-induced gelation of coil structure polysaccharides (e.g., κ-carrageenan), (**B**) ion-induced egg-box gelation of alginate, (**C**) covalent crosslinking-induced gelation (e.g., epichlorohydrin for cellulose hydrogel induction, glutaraldehyde for chitosan hydrogel induction), (**D**) pH-induced gelation (e.g., induction of pectin hydrogels) [42].

Polysaccharides, proteins, and polyphenols in food form multifunctional colloidal materials through covalent or non-covalent interactions, demonstrating extensive application potential in the food industry [9]. The formation of polysaccharide hydrogels can be induced by various methods, including heating and cooling, pH and salt adjustments, the addition of sucrose, and freeze–thaw cycles [21]. Polysaccharide colloids such as sodium alginate increased nutraceutical stability and bioaccessibility [22] and guar gum enhanced the pasting stability and gel properties of pea starch gels [23,24]. Polysaccharides play a crucial role in plant-based foods due to their unique gelling and thickening properties. Other polysaccharides like carrageenan [25,26], konjac glucomannan [27], chitosan [28,29], alginates [30,31], inulin [32,33], starch [34], cellulose [35], gum arabic [36,37,38,39], and gellan gum [40,41] form gels through various mechanisms, as illustrated in Figure 1. These colloids enhance the water-holding capacity of food, improve mouthfeel, and provide stable food structures.

As core functional components of food colloidal systems, polysaccharides not only play a key role in constructing food matrix structures, improving their texture and sensory characteristics and extending their shelf life, but also offer extensive application potential for developing biomimetic plant-based foods with superior functional properties through their unique thickening, gelling, and emulsifying characteristics [43]. In particular, plant polysaccharides as fat replacers can significantly enhance the water-holding capacity, texture, and rheological properties of myofibrillar protein gels and improve gel characteristics under low-salt conditions, thereby increasing the nutritional value and sensory quality of meat products [44].

Studies have found that the addition of plant polysaccharides such as inulin, κ-carrageenan, and konjac glucomannan as fat replacers to myofibrillar protein gels can significantly enhance the water-holding capacity, texture, and rheological properties of the gels. The incorporation of 1% konjac glucomannan can significantly increase water-holding capacity and gel strength, effectively restricting water mobility, demonstrating great potential as a fat replacer in meat products, even with reduced digestibility of the myofibrillar protein [10]. The insoluble dietary fiber from shiitake mushroom stems can significantly improve the gelling performance of myofibrillar protein, such as water-holding capacity and gel strength, and enhance the nutritional value and sustainable resource utilization of low-fat meat products by influencing water distribution, microstructure, and intermolecular interactions, contributing to the dual development of functionality and environmental friendliness in meat products [45]. Potato dietary fiber improves the structure and thermal aggregation behavior of myofibrillar protein by increasing the content of α-helices and β-sheets and reducing the crosslinking of aliphatic residues; thereby, it increases the particle size, turbidity, and surface roughness of myofibrillar protein, decreases the zeta potential, accelerates the gelation rate, and significantly enhances the elasticity, viscosity, strength, and water-holding capacity of gels, ultimately forming a more uniform and compact microstructure and effectively improving the quality of processed chicken patties [46]. Plant polysaccharides have the potential to enhance the quality of low-salt meat products. Research has found that konjac glucomannan improves the gel characteristics of low-salt myofibrillar protein by modifying protein conformation. Konjac glucomannan significantly enhances the gel characteristics of myofibrillar protein under low-salt conditions by altering the conformation of myofibrillar protein and enhancing the binding ability of troponin-T to myosin, including increasing the storage modulus (G′) and gel strength of thermally induced gels, providing effective molecular mechanisms and theoretical basis for improving the quality of low-salt meat products [47]. Furthermore, research has discovered that the addition of 5.0%~7.5% quinoa protein improves the intermolecular interactions and structural stability of myofibrillar protein gels and enhances the gelling and water-holding properties of myofibrillar protein. The addition of quinoa protein significantly improves the physical properties and structural stability of myofibrillar protein gels by increasing the content of disulfide bonds, promoting the formation of hydrogen bonds, and enhancing hydrophobic and electrostatic interactions. In this way, it leads to a transition in the protein structure from α-helices to β-folds, thereby enhancing the gelling and water-holding properties of myofibrillar protein, providing potential application strategies for developing novel fish products, and improving the quality of meat products [48].

Interactions between different ionic types of polysaccharides and myofibrillar protein significantly impact the digestibility and nutritional release of gel-based foods. A simulated gastrointestinal digestion study was conducted using anionic xanthan gum (XMP) and sodium alginate (SMP)/cationic chitosan (CSMP)/neutral casein (CMP) and konjac (KMP) as materials to prepare high-quality gel foods before and after consumption. The results indicated that interactions between different ionic types of polysaccharides (anionic, cationic, neutral) and myofibrillar protein (MP) significantly affect the digestibility and nutritional release of MP gels, where neutral polysaccharides enhance gel strength and protein digestibility while cationic polysaccharides, despite increasing gel strength, inhibit protein hydrolysis [49].

These findings provide a scientific basis and regulatory strategies for developing gel foods with good quality and digestibility by controlling the ionic types of polysaccharides, contributing to the nutritional, health, and sustainable development of food.

### 2.3. Composite Plant-Based Food Colloid Systems

**Figure 2:**
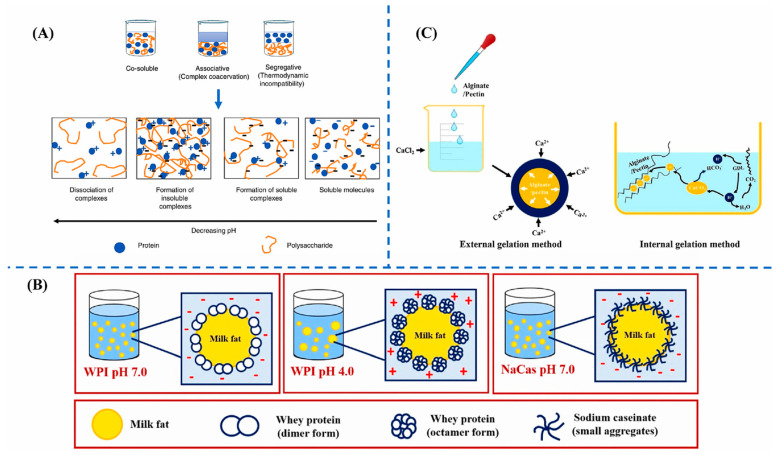
Food gel component interoperation mode. (**A**) Polysaccharide and protein interactions. (**B**) Protein and lipid interactions. (**C**) Polysaccharide and Ca^2+^ interactions [50].

Composite colloid systems combine the characteristics of plant protein and polysaccharide colloids to achieve superior functionality and sensory properties. The design concept of such systems is to create plant-based foods with better texture and structure by complementing the performance of different colloids. Plant-based foods are typically composed of various components, including proteins, polysaccharides, and lipids. By utilizing the interactions between different components, composite food colloid systems can be constructed, as shown in Figure 2, thereby achieving a synergistic improvement in the quality of plant-based foods [42].

Research has found that soy protein isolate (SPI)–naringin (Nar) complex pre-aggregates form gels through ohmic heating (OH) and the addition of chitosan (CS). When the amount of added CS is 3%, the gel (SPI-Nar-CS 3) exhibits the best rheological properties, with a hardness, elasticity, and water-holding capacity of 90.67% [51]. Studies have discovered that under optimal conditions, ionic polysaccharides (including chitosan, alginates, carrageenan, and xanthan gum) can improve the functional properties of soy protein by altering its secondary structure; relaxing its structure will expose the hydrophobic regions of the protein more, making it suitable for wide applications in the food industry [52].

Overall, the application of food colloids in biomimetic plant-based foods mainly focuses on improving the sensory quality of plant-based foods. Moreover, research on the role of food colloids in enhancing the sensory quality of plant-based foods primarily concentrates on the following aspects:

Utilizing plant-based particles as stabilizers can construct stable Pickering emulsions, which can simultaneously contain lipophilic and hydrophilic bioactive compounds, thereby improving the functionality of plant-based products [53,54]. In the construction of plant protein-based Pickering emulsions, modifying plant proteins can effectively enhance the sensory quality of plant-based foods [55].

Food gels are typically composed of biopolymer molecules or colloidal particles dispersed in water and can be used for encapsulating and releasing bioactive substances. Through enzymatic synergy, the performance of food gels can be further enhanced, thus improving the sensory quality of plant-based foods. Additionally, a study compared the effects of transglutaminase and laccase on the properties of pea protein gels. The results showed that both enzymes could enhance the performance of pea protein gels, with transglutaminase exhibiting particularly outstanding performance in increasing gel strength and water holding capacity [56].

Pectin, as a natural polysaccharide, can form a three-dimensional hydrophilic polymer network, providing softness, flexibility, and biocompatibility to hydrogels. These gels can be cross-linked through physical, chemical, and interpenetrating polymer network methods, aiding in the encapsulation of bioactive substances and improving their stability and controlled release [57].

Gels, as soft and elastic polymeric materials, can retain large amounts of liquid within their three-dimensional network structure, providing an ideal platform for encapsulating bioactive substances [58]. Protein-based emulsion gels have been explored for their potential as delivery materials for bioactive substances. These emulsion gels can provide a stable network structure, improve the physicochemical stability and texture of substances, and offer new possibilities for the stable loading and controlled release of functional substances [59].

Colloid delivery systems assembled from plant-based components, such as lipids, proteins, polysaccharides, phospholipids, and surfactants isolated from plant sources, create plant-based nanoemulsions, nanoliposomes, nanoparticles, and microgels. These delivery systems encapsulate, protect, and release various bioactive substances, including oil-soluble vitamins (such as vitamin D), novel omega-3 oils, carotenoids (vitamin A precursors), curcumin, and polyphenols [60].

Food colloids play a crucial role in the design of biomimetic foods, not only influencing the sensory properties of foods but also having a significant impact on food stability, nutrient release, and health benefits. Composite plant-based food colloid systems can harness the synergistic functions of multiple components, providing new ideas for designing novel biomimetic plant-based foods. Future research should continue to explore the intricate relationship between the structure and function of food colloids and their behavioral changes under different processing conditions, thereby providing a theoretical basis and technical support for developing higher-quality biomimetic plant-based foods.

## 3. Application of Food Colloids in Biomimetic Animal-Based Foods

With increasing concerns about health, sustainability, and ethics, consumers are becoming more inclined to reduce their intake of animal-based foods. For this reason, the food industry is creating new products using plant-based ingredients to mimic many of the physicochemical and sensory properties associated with animal-sourced foods (including seafood analogs, dairy analogs, egg analogs, and meat analogs) [61]. Animal-based foods are highly favored by consumers due to their unique flavors and textures. However, the production process of traditional animal-based foods often faces problems such as high resource consumption and severe environmental pollution. Food colloids provide new ideas and methods for developing sustainable biomimetic animal-based foods. This section will focus on exploring the application of food colloids in biomimetic animal-based foods, mainly covering three aspects: milk-based food colloid systems, meat-based food colloid systems, and egg-based food colloid systems.

### 3.1. Application of Food Colloids in Biomimetic Milk-Based Food Colloid Systems

**Figure 3:**
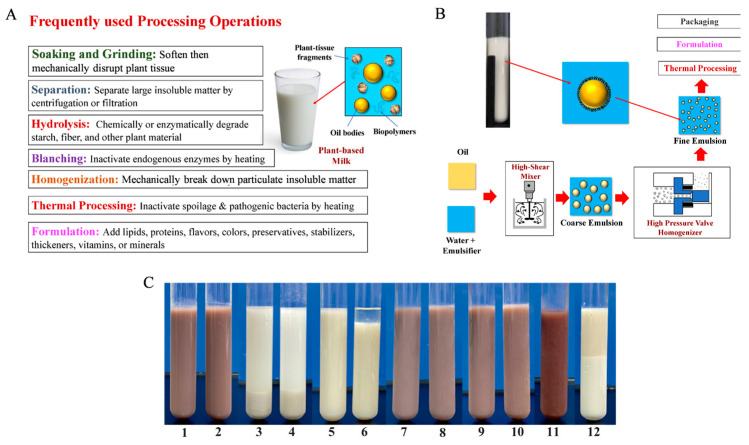
(**A**) Some commonly used processing operations that are utilized to produce plant-based milk substitutes. (**B**) Plant-based milk substitutes can also be produced by homogenizing plant-based oil and emulsifiers with water. Typically, a coarse emulsion is formed first, which is then passed through a homogenizer [64]. (**C**) Plant-based milk substitutes: (1) adzuki bean milk (homogenized); (2) adzuki bean milk (unhomogenized); (3) adlay milk (homogenized); (4) adlay milk (unhomogenized); (5) oat milk (homogenized); (6) oat milk (unhomogenized); (7) adzuki bean–adlay milk (homogenized); (8) adzuki bean–adlay milk (unhomogenized); (9) adzuki bean–oat milk (homogenized); (10) adzuki bean–oat milk (unhomogenized); (11) adzuki bean–adlay milk (commercial); (12) oat milk (commercial) [65].

In recent years, the market demand for plant-based milk alternatives has been rising due to concerns about the healthiness of cow’s milk, such as lactose intolerance and hypercholesterolemia. Another reason is the change in lifestyles, such as vegetarianism [62]. The formulation and manufacturing process of plant-based milk includes the formation of oil dispersions by mechanically breaking down plant materials or the formation of oil-in-water emulsions through homogenization [63]. Generally, two main methods have been developed to produce these products. Figure 3A shows the first method, which is breaking down the natural structure of certain plant materials to release oil bodies or other colloidal substances; Figure 3B illustrates the second method, which is using plant ingredients to manufacture simulated fat globules [64]. These plant-based products mimic dairy products such as milk, cheese, and yogurt. They are typically made from nuts, soybeans, chickpeas, oats, seeds, cassava, yeast, etc. [62].

In the development of plant-based dairy products, technological innovation and optimization of production processes are key to improving product quality. He and Xu pointed out through research that using high-pressure homogenization technology can significantly improve the physicochemical and sensory properties of plant-based milk based on adzuki bean, adlay, and oat, as shown in Figure 3C [65]. This technology reduced the particle size by more than 50% by increasing the brightness and Brix value of the samples and significantly improved their viscosity. Yao et al. explored the physicochemical and sensory characteristics of six plant-based milk alternatives (soybean, peanut, adlay, adzuki bean, oat, and buckwheat) under different grain-to-water ratios and found that all types of plant-based milk, except for buckwheat milk alternatives, had stable hydrophilic colloid systems, and the aroma and taste of the products enhanced consumers’ overall love for the products [66].

Food colloids play a crucial role in improving the stability and sensory properties of plant-based milk. Xiong et al., in a review of the application of 12 types of legumes (such as soybean, broad bean, mung bean, etc.) in plant-based milk alternatives, emphasized the potential of food colloids in addressing defects such as nutritional imbalance, off-flavors, and emulsion stratification [67]. Food colloids can not only act as emulsifiers and thickeners but also improve the texture and mouthfeel of milk alternatives. The nutritional composition of plant-based milk products is key to assessing their value as dairy alternatives. Walther et al. quantitatively and qualitatively analyzed the protein, carbohydrate, fat, vitamin, and mineral content, as well as pesticide residues, in 27 different types of plant-based beverages and two cow’s milk samples [68]. The research results showed that most plant-based beverages were deficient in nutritional composition compared to cow’s milk, especially in terms of protein content and quality, highlighting the importance of improving the nutritional balance of plant-based beverages. More importantly, plant-based synthetic dairy products usually do not contain lactose or animal protein. This is an advantage for lactose-intolerant individuals, who will not experience digestive problems such as bloating and diarrhea due to lactose intake. For people who are allergic to milk protein or animal dairy products, synthetic dairy products are a safe alternative. In addition, the production of plant-based synthetic dairy products usually relies on plant-based raw materials such as soybeans, which can reduce greenhouse gas emissions during cultivation and production and promote the development of sustainable agriculture [69,70].

The health benefits of plant-based milk products are another factor driving the growth of their market demand. Hidalgo-Fuentes et al. discussed how fermentation processes can improve the sensory properties, nutritional value, and functional/bioactive properties of plant-based beverages [71]. The fermentation process can enhance flavor compounds and improve the digestibility and bioavailability of nutrients while reducing the presence of anti-nutritional factors and providing consumers with beverages that have multiple bioactivities, such as antioxidant, anti-inflammatory, and hypotensive effects.

Food colloids can exert thickening, gelling, and emulsifying functions, which are key to improving the texture and stability of biomimetic dairy products. Research on biomimetic cheese and dairy products is relatively scarce, and their structure and flavor are difficult to simulate. Traditional soy cheese and tofu have a monotonous texture and differ greatly from dairy products. In addition to colloids, ingredients such as starch, nut oils, and plant proteins are also important raw materials for biomimetic dairy products [72]. Food colloids have a synergistic effect with these components and jointly shape product characteristics. To address the problems of bland flavor and rough texture in biomimetic dairy products, their sensory quality can be improved by screening suitable food colloid matrices, optimizing the ratio of colloids to other ingredients, and introducing fermentation processes.

### 3.2. Application of Food Colloids in Biomimetic Meat-Based Food Colloid Systems

Fat in meat products plays a vital role in enhancing sensory properties. However, the long-term consumption of saturated and trans fats can lead to an increased risk of metabolic diseases such as coronary heart disease, cardiovascular disease, atherosclerosis, type II diabetes, and obesity. As excessive meat consumption poses health hazards, there is a growing trend in the consumption of plant-based meat and fat substitutes [10]. Plant-based simulated animal foods mainly include dairy analogs, meat product analogs, seafood analogs, and egg analogs [73,74,75].

The manufacturing of artificial meat primarily involves two technologies: plant-based artificial meat and cell-cultured meat. Simulating the texture and mouthfeel of meat through the structured processing of plant-based polymers has become a popular processing method. The main types of plant-based polymer colloids used as meat fat substitutes include emulsions, hydrogels, and oleogels. Plant-based artificial meat is favored by vegetarians. Animal food substitutes can be prepared through various methods, such as extrusion, shear cell technology, 3D printing, shearing–gelling, wet spinning, and freeze-structuring [76,77,78]. Imran and Zhang provided a comprehensive review of the manufacturing methods for plant-based meat [79]. Another option is novel cultured meat or “lab-grown meat,” which can provide a rich source of protein. The application of cellular agriculture in plant-based meat analogs can improve their sensory properties and reduce their environmental impact [80].

#### 3.2.1. Cell-Cultured Meat

**Figure 4:**
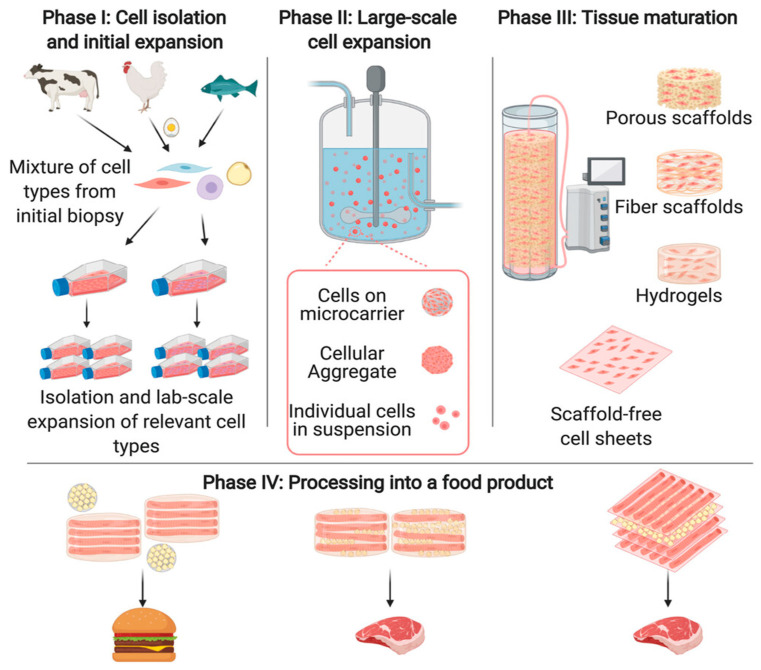
The process of cell-cultured meat [89].

Cell-cultured meat utilizes the method of animal cell tissue culture to form a whole piece of artificial meat through the proliferation, differentiation, and fusion of muscle stem cells or muscle satellite cells (Figure 4) [74]. The production of cultured meat requires appropriate tissue engineering techniques to construct 3D edible muscle tissue, including the use of suitable cells and manufacturing technologies to simulate complex natural tissue. Common alternatives to animal-derived scaffold agents can be materials derived from algae, plants, and bacteria, as well as recombinant bioactive materials (e.g., recombinant collagen or gelatin) [81,82,83]. Food colloids, due to their safety and thermal stability, as well as their ideal taste, texture, and nutritional qualities when consumed, have obvious advantages in scaffold materials for cell-cultured meat. Soy protein [84], wheat protein [85], pea protein [86], carrageenan [87], and chitosan [88] serve as smart 3D muscle cell culture scaffolds in food colloids.

One study evaluated two plant protein-fortified scaffolds (a blend of pea protein isolate and soy protein isolate with RGD-modified sodium alginate) as a 3D printing platform for bovine satellite cell (BSC) maturation. The experimental results demonstrated that the mold-based plant protein scaffolds outperformed the single sodium alginate scaffold in terms of stability and stiffness, supporting the effective expansion and maturation of BSCs. Furthermore, by employing extrusion-based 3D printing technology, combined with an edible removable agar support bath, constructs with clear geometries were successfully fabricated, effectively supporting BSC attachment and differentiation. Moreover, cell bioprinting using pea protein-enriched bioinks exhibited cell viability up to 80–90%, and cells were capable of maturing within the 3D-printed constructs [81].

The roles of hydrogels in cell-cultured meat include serving as a soft 3D ECM-like environment [90,91], acting as a 3D matrix filler inside porous scaffolds [92], being a components of bioinks [93], functioning as thin membranes which may be microstructured to produce the alignment of cells [94], or being the source material to develop porous scaffolds [87]. Hydrogels mainly play a role in the first three applications, where solidification can be achieved through enzyme gelation, thermal gelation within the cell-compatible temperature range (4–37 °C), photopolymerization using cell-compatible exposure durations and wavelengths, or ionic crosslinking gelation. Compared to hydrogels composed of a single material, composite hydrogels can better summarize the ECM and often exhibit better performance. Hyaluronic acid and collagen composites more accurately summarize the ECM and are used for 3D adipogenesis. Similarly, collagen–fibrin composites provide short-term (fibrin) and long-term (collagen) ECM, making this composite more suitable for tissue maturation. Hyaluronic acid and alginate composites improve gel properties compared to using hyaluronic acid alone [89].

#### 3.2.2. Plant-Based Meat Alternatives

Plant protein technology is advancing the development of plant-based meat alternatives through continuous technological innovations, such as fiber spinning, extrusion, shearing, and 3D printing production processes. These processes aim to simulate the appearance, color, texture, and mouthfeel of animal meat, catering to the growing global consumer demand [78].

Developing meat alternatives using legumes and plant-based ingredients can contribute to improved sustainability in food production and may offer health benefits [95]. Julie et al. developed a protein based on blue-green algae, which exhibits a texture and mouthfeel similar to real meat. Blue-green algae grow through photosynthesis, require minimal processing, and can efficiently produce protein fibers [96]. Fungal protein, as an emerging alternative protein, has a balanced and comprehensive amino acid composition, is rich in dietary fiber, and has a low saturated fatty acid content. The fibrous structure of the fungal mycelium can effectively replicate the texture of meat, showing great potential for developing meat alternatives. A research progress review pointed out that fungal protein has an immense potential in developing meat analog products due to its balanced and comprehensive amino acid composition, abundant dietary fiber, and low saturated fatty acid content. The fibrous structure of the fungal mycelium can effectively replicate the texture of meat, making it a focus of attention in the alternative protein field [97]. Soy protein is one of the most widely used plant proteins in the market currently. By adding new ingredients such as fungal protein and soy hemoglobin, the quality (i.e., texture and flavor) of plant-based meat can be significantly improved [98].

**Figure 5:**
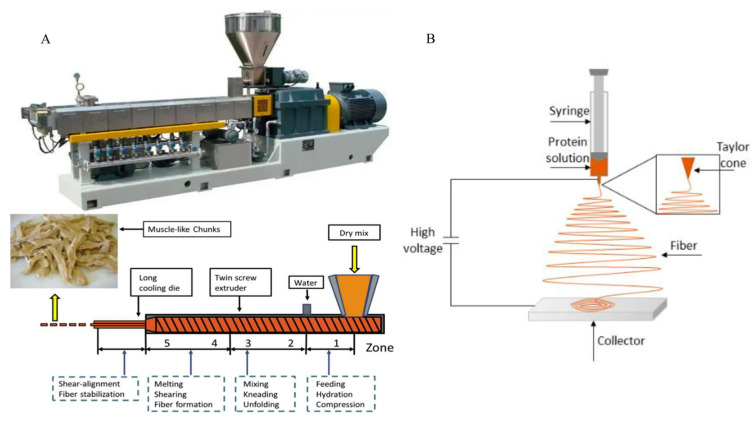
(**A**) High-temperature hot extrusion of protein into meat analogs [16]. (**B**) Electrospinning [99].

The preparation techniques for plant-based meat alternatives include mechanical structuring techniques for plant-based polymers and colloidal structuring techniques for meat fat substitutes. Mechanical structuring techniques include extrusion (Figure 5A), shearing, structured freezing, and 3D printing [16]. Colloidal structuring techniques include hydrogels, emulsions (emulsions and emulsion gels), and oleogels. In addition to extrusion technology, colloidal structure design techniques such as electrospinning, directional freezing, directional stretching, and double network gels can also be applied to the production of plant-based artificial meat. These techniques simulate the multi-level anisotropic structural characteristics of real meat, improving the textural fidelity of artificial meat [2]. Colloids can also enhance the solubility, water retention, and emulsification properties of proteins, improving the processing performance and sensory quality of biomimetic meat.

The most mature mechanical structuring method for plant-based meat is hot extrusion technology. Hot extrusion technology typically requires ultra-high-temperature treatment of raw materials, which significantly improves the formation of fibrous structures in plant-based materials but is detrimental to the retention of nutrients. Three-dimensional printing technology is relatively mature, operates at low processing temperatures, and can produce customized shapes. However, unlike extrusion technology, it cannot be applied to large-scale production. Freeze structuring technology and Shell Cell technology are relatively new but not mature enough; they are currently in the experimental stage [100]. However, compared to extrusion technology and 3D printing technology, these two technologies have milder conditions for preparing plant-based meat alternatives. In large-scale production, the cost of freeze structuring technology may be higher than extrusion due to the high energy consumption of freeze-drying. The volume of Shell Cell equipment is smaller than extrusion equipment, and it can only prepare small quantities of plant-based meat products. By increasing its sample processing capacity, its application scale in the plant-based meat industry can be expanded.

These plant-based products mimic the taste, texture, and nutritional profile of meat. They are typically made from soy, wheat, or pea protein and can be prepared in various forms, such as burgers, sausages, and meatballs.

#### 3.2.3. Fat Substitutes

Applying plant-based polymers to fat substitutes through colloidal structuring technology is a more flexible processing method, which is mainly influenced by the gelling properties and pretreatment of plant proteins and polysaccharides themselves [61]. Emulsion systems and oleogel systems have very high requirements for the interfacial stability of plant-based polymers. Moreover, since the oils used in these two colloidal systems usually contain a higher proportion of unsaturated fatty acids, plant-based polymers are also required to have better antioxidant properties. To meet the demand of consumers for fat substitutes, extensive research is still needed to expand the properties of plant-based polymers for flexible application in various colloidal systems. Plant-based meat and meat exhibit different protein digestion rates and peptide/amino acid compositions during the digestion process, which is closely related to the structure and physicochemical properties of different types of plant proteins; at the same time, the processing conditions during mechanical structuring also significantly affect the structure and physicochemical properties of plant proteins [16,101].

To address issues such as the weak gelling ability and poor mouthfeel of plant proteins, the quality of biomimetic meat can be improved by compounding different food colloids, optimizing processing techniques, and regulating sensory properties. For example, high-shear and high-pressure treatments can be used to enhance the interaction between proteins and colloids, 3D printing can be employed to shape muscle texture, and the addition of spices and heme can improve flavor and color.

### 3.3. Egg-Based Foods

Eggs are a complex colloidal system composed of various proteins, lipids, and minerals. Based on the structure and properties of egg colloids, various biomimetic egg products can be developed. With the rise of plant-based diets, the demand for egg substitutes is growing. Traditional bean products, such as tofu eggs and chickpea eggs, differ significantly from real eggs in texture and flavor, and there is an urgent need to develop highly realistic biomimetic egg products. The thermal gelling property of eggs is one of their important functional characteristics. Food colloids such as gelatin and agar have similar heat-induced gelling properties, forming a three-dimensional network when heated and solidifying when cooled, which can be used to simulate the gelling process of eggs. Different food colloids play different roles in biomimetic egg products. For example, carrageenan gels have high transparency and can simulate egg white; konjac gum gels have good shear-thinning properties and can simulate the fluidity of egg yolk; gelatin gels have a delicate texture and a strong ability to combine with proteins, which can shape the composite texture of egg white and egg yolk. These plant-based products can replicate the taste and texture of eggs. They are typically made from tofu, chickpea flour, or water.

Research has found that emulsion gels containing 12.5 wt% RuBisCO and 10 wt% corn oils can mimic the nutritional composition of whole eggs. After forming an oil-in-water emulsion and heating it to transform it into an emulsion gel, the results showed that as the oil droplet size decreased, the brightness and hardness of the gel increased, allowing the appearance and texture to be regulated by controlling the oil droplet size. Furthermore, after incorporating different concentrations of curcumin (3, 6, and 9 mg/g oil) into the emulsion using a pH-driven method, curcumin acted as a natural bifunctional ingredient (colorant and nutritional component), and its yellow-orange color made the appearance of the emulsion gel match that of raw and cooked whole eggs. Plant-based emulsion gels can be combined with natural pigments to successfully prepare whole egg substitutes [102].

A study on the freeze–thaw stability and thermal gelling properties of mung bean protein-based emulsions through pH shifting and transglutaminase (TG) cross-linking found that two different oil phases (rapeseed oil and coconut oil) were used in the preparation. The study showed that pH shifting could significantly improve emulsifying activity (up to a 77.6% increase) and reduce oil droplet size (from 39 μm to 7 μm). After freeze–thaw treatment, the emulsion samples prepared with pH-shifted mung bean protein (BMBP), regardless of whether they underwent TG cross-linking or were in different oil phase systems, exhibited better freeze–thaw stability than native mung bean protein (NMBP). Moreover, the apparent viscosity of BMBP emulsions was higher than that of NMBP, and the viscosity of BMBP-based coconut oil emulsions before freeze–thawing (222.50 mPa·s) was closest to that of real liquid eggs (184.86 mPa·s). In particular, BMBP was more sensitive to TG, resulting in better thermal gel hardness in BMBP-based emulsions [103].

Amaranth protein isolate (API) was extracted from amaranth using the alkaline extraction/acid precipitation method. The results showed that API had good water and oil absorption capacity, superior emulsifying ability, and significantly higher emulsion stability at pH 2.0. Five different API/EY combination ratios (0/0.75, 0.25/0.5, 0.375/0.375, 0.5/0.25, 0.75/0) were prepared in the study. The color, emulsion stability (ES), freeze–thaw stability (FTS), oil droplet size, structure, rheological properties, and sensory characteristics of the samples were evaluated. Low concentrations of API (0.25% and 0.375%) significantly increased the oil droplet size and decreased the FTS of low-fat mayonnaise (LFM) emulsions. The sample containing 0.75% API had the significantly highest zero-shear viscosity (η₀) and relaxation time (λ). In summary, API can be a suitable substitute for EY in LFM production, has the potential to promote human health, and provides a new strategy for preparing plant-based products [104].

In a study, 93 participants completed a survey on plant-based eggs and evaluated different food samples (including traditional chicken eggs and scrambled tofu) using a hedonic scale, check-all-that-apply (CATA), and temporal check-all-that-apply (TCATA) while recording their emotional responses and intended use of plant-based eggs (PBEs). The results showed that participants expressed interest in plant-based alternatives (including PBEs) but had concerns about their sensory characteristics. Compared to chicken eggs, participants rated the taste and texture of PBEs lower, mainly due to the beany, bitter, and off-flavor attributes of PBEs. PBEs were also associated with negative emotions, while the liking of scrambled tofu did not differ significantly from that of chicken eggs, and both were mainly associated with positive emotions. In the TCATA assessment, participants focused on the flavor attributes of PBEs, while their evaluation of chicken eggs focused on texture attributes. Although consumers showed interest in PBEs regardless of whether they followed a plant-based diet, the sensory characteristics of PBEs need improvement for consumers to be willing to incorporate them into their diets [105].

### 3.4. Application of Food Colloids in Biomimetic Fish and Seafood Products

The application of food colloids in plant-based fish and seafood analogs is primarily designed to mimic the flavor, texture, and nutritional characteristics of fish [99,106]. Mimicking the internal structure and texture of fish or seafood requires simulating their nanofibrous structure, partially or entirely replacing fish raw materials or fish myofibrillar proteins, mainly derived from plant protein isolates or concentrates incorporated into fish-quality gels [107]. These plant-based products are typically made from algae, seaweed, soy protein, and gluten, and can be used in various dishes such as sushi, crab cakes, fish and chips. To achieve a meat-like structure, plant-derived ingredients undergo multiple processing steps, including heat treatment (high-temperature cooking or extrusion) [108]. Whole products such as proteins, carbohydrates, lipids, and legumes can be used to produce plant-based fish alternatives. Soybeans, wheat, peas, rice, corn, potatoes, as well as other algae, sunflower, and rapeseed proteins, are the main sources for preparing biomimetic fish and seafood products [73,109].

Researchers have also conducted studies to obtain high-quality products using different food ingredients. For example, shrimp analogs were obtained from sunflower oil, alginate, egg white powder, sucrose, glycine, and CaCO_3_. Furthermore, combining shrimp analogs with coconut shell ethanol extract and bean curd extract extended the shelf life of ready-to-eat vegetarian products from 12 days to 21 days and 16 days, respectively, under refrigeration [110].

The techniques for processing seafood are similar to those for meat products; the most commonly used are organized plant proteins with a fibrous, meat-like structure, produced using an extrusion process. Less popular techniques in the food industry that may only be used for producing animal product analogs include electrospinning, wet spinning, directional freezing, and shear cell meat [73]. Other techniques used in the food industry are 3D and 4D printing. These technologies are much more costly and time-consuming, but they allow for personalized products. Less popular techniques in the food industry that may only be used for producing animal product analogs include electrospinning, wet spinning, directional freezing, and shear cell.

Using duckweed RuBisCO protein and omega-3-rich flaxseed oil, a simulated plant-based sea foie gras analog was developed, consisting of gels composed of high concentrations of flaxseed oil droplets dispersed in a gelled ruby protein network. It was hypothesized that the high dispersed phase volume fraction of oil droplets and the heat-stable gelling properties of ruby protein would enable us to generate sea foie gras analogs. Natural pigments (β-carotene) were incorporated into the oil phase of the emulsion to mimic the traditional orange color of analogs. Emulsion gels prepared with 40% flaxseed oil and 10% ruby protein produced plant-based products that closely mimicked the texture and color of the real product. Rheological analysis indicated that the oil droplets were active fillers in the protein gel [111]. A study on the effect of modified potato starch additives on the biochemical and structural properties of shrimp analogs/imitation shrimp prepared from fish liver as raw material found that during frozen storage (−20 °C), the 100% modified potato starch-added shrimp analog samples had the least myofibrillar protein loss. During long-term low-temperature storage, seafood analogs were greatly influenced by the type of starch added. The addition of modified potato starch improved the sensory quality and structural properties of shrimp analogs and reduced quality degradation during frozen storage [112].

Biomimetic fish and seafood products primarily use plant proteins such as soy protein and wheat gluten as the main raw materials, but they differ significantly from real aquatic products in terms of texture and mouthfeel [99]. Seaweed extracts such as alginate and carrageenan, which are food colloids, have similar gelling properties to fish meat and have received attention in the research of biomimetic aquatic products. For example, alginate can simulate the elasticity of fish meat, while carrageenan can simulate the chewiness of shrimp meat. The combination of the two can shape the texture of biomimetic fish paste [106].

The combination of food colloids and plant proteins is an effective way to improve the quality of biomimetic aquatic products. For example, the combination of soy protein isolate and carrageenan can increase gel strength and water retention; the combination of pea protein isolate and gelatin can improve emulsion stability; and the combination of wheat gluten and sodium alginate can enhance texture and chewing sensation.

To address the issues of monotonous flavor and soft texture in biomimetic aquatic products, various food colloid combinations, the optimization of processing parameters, and the regulation of flavor and texture can be employed to improve quality. For instance, using a combination of alginate and fish collagen, along with a low-temperature slow-cooking process, can simulate the connective tissue between the myosepta of salmon; adding marine fish extracts and chitosan can enhance umami and seafood flavors [73].

## 4. Application of Food Colloids in 3D-Printed Biomimetic Foods

Three-dimensional-printed food is an emerging food processing technology that enables personalized customization and precise nutrition through digital design and precise control. Three-dimensional or four-dimensional printing allows for the preparation of personalized foods with complex structures, layer by layer, based on programmed digital models created using computer-aided design/manufacturing (CAD/CAM) software [113,114,115]. Food colloids play a crucial role in 3D=printed foods, not only influencing the rheology and formability of the printed food but also having a significant impact on the texture and sensory characteristics of the printed food [116]. In the field of food technology, 4D printing is an emerging and relatively unknown technology. Four-dimensional printing is an additive manufacturing technology and an extension of three-dimensional printing. Moreover, 4D printing is similar to 3D printing in terms of design development and printed structures with 3D printers [117,118]. The main difference between 4D and 3D printing is intelligent design and smart materials, as 4D-printed structures can change shape or function [119,120]. Food can change color, texture, taste, and shape when subjected to various stimuli, such as temperature and acidity. Therefore, 4D printing saves space during the transportation process or adjusts the printed materials according to customer needs.

This section will focus on exploring the application of food colloids in 3D-printed biomimetic foods, mainly covering three aspects: the food colloid characteristics of food-grade inks, the influence of colloid rheology on 3D printing performance, and typical 3D-printed biomimetic foods and their colloid characteristics.

### 4.1. Food Colloid Characteristics of Food-Grade Inks in 3D Printed Biomimetic Foods

Food-grade inks refer to the edible raw materials used for 3D food printing, usually consisting of a mixture of food components and additives. The colloidal properties of food-grade inks, such as rheology, stability, and formability, are critical factors affecting the quality of 3D printed foods.

Additives play a crucial role in improving the performance of food colloids. Wang et al. found that adding polysaccharides and chitosan to Pickering emulsions significantly improved the rheological properties and mechanical performance of the emulsions, thus enhancing their suitability as 3D printable edible inks [121]. Sun et al. investigated a kelp gum-based food 3D printing ink and discovered that it exhibited good 3D printability when combined with colloidal and protein/polysaccharide/starch substances. This kelp gum-based food 3D printing technology can significantly improve the precision and stability of printed foods while also providing rich nutritional components [122].

The optimization of sensory characteristics is crucial for improving consumer acceptance of printed foods. The elderly, due to swallowing difficulties and reduced chewing ability, require soft-textured and easy-to-swallow foods. Yun et al. developed a 3D printable food ink containing abalone powder, adjusting its texture by adding gelatin to meet the swallowing needs of the elderly [123]. Furthermore, Zhong et al. investigated the application of egg yolk powder-starch gel as a novel 3D-printable food ink and found that it possesses high storage modulus and printing accuracy [124].

These studies demonstrate that the colloidal properties of food-grade inks are closely related to their 3D printing performance, and optimizing the ink formulation can effectively improve the quality of printed foods.

### 4.2. Influence of Colloid Rheology on 3D Printing Performance

Rheological properties are crucial factors affecting the performance of food colloids during the 3D printing process. Chen et al. pointed out that suitable rheological properties are the basic conditions for achieving successful printing. Therefore, many studies focus on improving the rheological properties of natural food colloids through additives, including lipids, colloids, and carbohydrates [125]. For example, Rahman et al. investigated the rheological and mechanical properties of agar and konjac gum at different ratios and found that increasing the content of konjac gum can effectively enhance the rheological performance, thus improving its applicability in extrusion-based 3D printing [126]. Belova et al. enhanced the texture and digestibility of κ-carrageenan food colloids by adding callus of Trigonella foenum-graecum [127]. By incorporating different types of colloids, such as xanthan gum, guar gum, and carrageenan, the mechanical properties and rheological behavior of food colloids can be significantly improved, thereby enhancing their suitability in the 3D printing process. The main mechanism of action of colloidal additives lies in their ability to interact with other components in food colloids, forming stable network structures. Wang et al. demonstrated that by adjusting the type and concentration of carbohydrates, the rheological properties of food colloids can be optimized, thereby improving their printing accuracy and stability [121]. Moreover, carbohydrate additives can also enhance the nutritional value and sensory characteristics of printed foods, making them more appealing to consumers. Drozdov and Christiansen modeled the rheological properties of plant protein–polysaccharide composite hydrogels and emulsions, revealing the influence of composition on their mechanical performance. By adjusting the ratio of plant proteins and polysaccharides, the rheological properties of food colloids can be significantly improved, thus enhancing their printability and stability [128].

In addition to rheological properties, formulation optimization is also an important means to improve the printing performance of food colloids. Wang et al. (2018) explored the formulation optimization of fish paste and found that an appropriate amount of sodium chloride can significantly improve its flowability and shape retention ability [129]. Furthermore, Yang et al. investigated lemon juice gel and optimized its rheological properties and mechanical performance by adjusting the addition of potato starch, making it more stable during the printing process to meet the requirements of 3D printing [130]. These studies demonstrate that by adjusting the proportion of ingredients, the printing performance of food colloids can be significantly improved.

Starch-based composite gels also have important application potential in 3D printed foods. Cui et al. investigated the rheological properties of normal corn starch and sodium alginate composite gels and found that the addition of sodium alginate can significantly enhance the printability of the composite gels [131]. The study also indicated that there is a close correlation between rheological properties and the printing window, and appropriate rheological properties can ensure the smooth progress of printing and the shape retention of the finished product. Moreover, the researchers employed computational fluid dynamics simulations to further explore the flow behavior during the printing process, providing a theoretical basis for optimizing the 3D printing of starch-based composite gels.

Improving mechanical properties is crucial for ensuring the structural integrity of printed products. Liu et al. studied the rheological and mechanical properties of milk protein composite gels and found that high concentrations of protein can improve their printing performance and ensure good morphological stability of the printed products [132]. Meanwhile, In et al. identified materials for 3D printed gel foods and emphasized the influence of rheological parameters on the fidelity, shape retention, and extrudability of the finished products [133].

The success of food printing is closely related to the rheological properties of the materials. Wedamulla et al. explored the influence of different formulations on the 3D printing gels of potato starch and found that the addition of pectin can significantly improve the printing performance, thereby enhancing the printing accuracy and structural stability [134]. Furthermore, Zheng et al. highlighted the relationship between rheological performance and printability, indicating that zero-shear viscosity and Young’s modulus are linearly related to printing height, providing a basis for optimizing printing parameters [135]. By adjusting the rheological properties of materials, more complex printing shapes and structures can be achieved. Feng et al. investigated the rheological properties of different ingredients (such as proteins and starches) during the 3D printing process and found that increasing the concentration of starch significantly improved the rheological performance, making it more suitable for 3D printing [136].

In summary, rationally regulating the rheological properties of colloids is crucial for achieving high-quality 3D printed foods. The rheological properties of colloids are closely related to their 3D printing performance. By optimizing the thixotropy and shear-thinning behavior of colloids, the quality of 3D-printed foods can be significantly improved. Through research on the rheological properties, formulation optimization, and mechanical performance of natural food colloids, numerous scholars have provided new ideas for enhancing the performance of food materials in 3D printing. These improvements not only promote the application of 3D printing technology in the food industry but also lay the foundation for developing more personalized and functional food products.

### 4.3. Three-Dimensional-Printed Biomimetic Foods and Their Colloidal Characteristics

Food colloids play a crucial role in 3D food printing technology due to their unique physical and chemical properties that can influence the rheology of printing materials, structural stability, and the quality of the final products. Understanding the characteristics of food colloids not only helps optimize the formulation of 3D printing materials but also improves the nutritional value and sensory properties of the final food products.

The application of food colloids in 3D printed foods has increasingly attracted the attention of researchers. Food colloids not only affect the rheological properties of printing materials but also directly relate to the texture and nutritional characteristics of the final products. Plant-based colloids, such as xanthan gum and konjac gum, are commonly used thickeners and gelling agents in food 3D printing. Jeon et al. demonstrated that xanthan gum can effectively improve the rheological properties of mixtures during the 3D printing process, thus supporting the printing of complex shapes [137]. Furthermore, Drozdov and Christiansen explored the rheological properties of plant protein–polysaccharide composite gels and found that their application in 3D printing inks exhibits good printability and stability [128]. These studies indicate that plant-based colloids can provide necessary mechanical properties and achieve different functional characteristics by adjusting the formulation.

Animal-derived colloids are equally important in 3D-printed foods. Feng et al. proposed that using Spirulina emulsion gels as printing materials can provide good rheological performance and impart nutritional value to the printed products [138]. Additionally, using whey protein as a base material for 3D printing inks has also shown good printability and texture of the final products. According to Huang et al., whey protein can form a stable colloidal network through heat induction during the 3D printing process, which is crucial for maintaining the structure of printed products [139].

Three-dimensional printing technology mainly includes extrusion printing, selective laser sintering, and inkjet printing methods. García-Segovia et al. investigated 3D printing based on xanthan gum and konjac gum and proposed key variables that influence printing conditions and characteristics, such as printing temperature and material formulation [140].

The structural characteristics of food colloids are another important aspect that affects their function and application. The microstructure of food colloids directly relates to their performance in the 3D printing process. Liu et al. studied the structural characteristics of different types of colloids in 3D printing and found that the stability of their microstructure is closely related to the quality of the final printed products [141]. For example, composite colloids using agar and gelatin have shown better self-supporting ability and shape retention, making them widely used in 3D printing.

Furthermore, Xu et al. explored how the network structure of different food colloids affects their performance in 3D printing, indicating that the density and distribution of the network structure have a direct impact on the stability of the printing results [142]. By regulating the crosslinking degree of colloids, their structural stability can be significantly enhanced, thereby improving printing accuracy and physical properties. In terms of the application of colloids, with the increasing demand for personalized and functional foods, the application range of food colloids is also expanding. Researchers are exploring how to use food colloids to manufacture personalized foods with special functions, such as foods for the elderly or patients with swallowing difficulties, infants and young children, weight loss groups, people with food component allergies, people with carbohydrate metabolism disorders (diabetic patients), lactose-intolerant individuals, athletes, etc. [143].

In summary, food colloids have an important application value in 3D-printed foods. Understanding their rheological and structural characteristics can provide theoretical support for improving printing materials and enhancing printing quality. Future research should focus on optimizing the formulation of these colloids to achieve better printability and final product quality. The types and characteristics of food colloids play a key role in the development of 3D-printed foods. Plant-based and animal-derived colloids have their unique advantages. Rationally combining and optimizing the use of these colloids can significantly enhance the functionality and sensory properties of 3D printed foods.

## 5. Conclusions, Challenges, and Prospects

Food colloid science provides an important theoretical basis and technical support for the research and development of biomimetic food. By revealing the intrinsic relationship between the structure and properties of food colloids, food colloid science can guide the selection of biomimetic food raw materials, the optimization of processing technology, and the improvement of product quality. At the same time, the research and development of biomimetic food also provides new opportunities and challenges for the development of food colloid science, promoting the continuous emergence of new technologies, methods, and theories in the field of food colloids. Therefore, there is a close relationship and mutual promotion between food colloid science and biomimetic food research.

Food colloids play an essential role in research on plant protein-based biomimetic meat products, dairy products, 3D-printed foods, seafood products, and egg products due to their unique physicochemical properties and functionality. By regulating the gelation properties, emulsifying ability, and stability of food colloids, the texture, mouthfeel, appearance, and taste of biomimetic foods can be effectively improved, providing new ideas for innovation in the food industry.

Although food colloid science has made significant progress in biomimetic food research, there are still many problems and challenges that need to be addressed. In the future, biomimetic food research should focus on breakthroughs and innovations in the following aspects:

(1) Developing new biomimetic food raw materials. In the future, more attention should be paid to the development and utilization of new biomimetic food raw materials, such as plant proteins, polysaccharides, and lipids, including pea protein, algal polysaccharides, and structured lipids, to obtain biomimetic food matrix materials with better quality and stronger functions.

(2) Exploring new biomimetic food processing technologies. New processing technologies such as 3D printing, electrospinning, and microfluidics provide new possibilities for the precise preparation of biomimetic foods. In the future, the application research of these new technologies in biomimetic food processing should be strengthened to achieve personalized customization and functional enhancement of biomimetic foods.

(3) Establishing new methods for evaluating biomimetic food quality. Sensory evaluation and instrumental analysis are the main methods to evaluate the quality of biomimetic foods, but traditional methods have limitations such as strong subjectivity and low efficiency. In the future, the integration of food sensory science with modern technologies such as machine learning and big data analysis should be strength more objective and efficient new methods for evaluating biomimetic food quality.

(4) Enhancing research on the safety and sustainability of biomimetic foods. While biomimetic foods bring convenience and enjoyment to consumers, their safety and sustainability cannot be ignored. In the future, research on the toxicology of biomimetic food raw materials, processing contamination, and the safety of new processes should be strengthened, and efforts should be made to improve the resource utilization efficiency and environmental friendliness of biomimetic food processing.

Although food colloids play an important role in biomimetic food research, they still face many challenges. These include the need for further research on the interaction mechanisms between food colloids and other components, the need for improvement in the processing technology and formula optimization of biomimetic foods, and the increasing consumer acceptance and awareness of biomimetic foods. However, with the increasing consumer demand for healthy, environmentally friendly, and sustainable foods, the market prospects for biomimetic foods are broad, providing opportunities for food technology innovation and industrial upgrading.

In the future, food colloids will continue to play an important role in the development of biomimetic foods. With continuous technological progress and in-depth research, the functionality of food colloids will be further expanded and optimized. Future biomimetic foods will be closer to the taste and nutritional characteristics of traditional animal foods while having higher health benefits, environmental friendliness, and sustainability. Food colloids will play an increasingly important role in the quality improvement, innovative product development, and market promotion of biomimetic foods.

## Data Availability

No new data were created or analyzed in this study.
